# Selection of Suitable Housekeeping Genes for Real-Time Quantitative PCR in CD4^+^ Lymphocytes from Asthmatics with or without Depression

**DOI:** 10.1371/journal.pone.0048367

**Published:** 2012-10-24

**Authors:** Ting Wang, Zong-An Liang, Andrew J. Sandford, Xing-Yu Xiong, Yin-Yin Yang, Yu-Lin Ji, Jian-Qing He

**Affiliations:** 1 Department of Respiratory Medicine, West China Hospital of Sichuan University, Chengdu, Sichuan Province, People’s Republic of China; 2 The UBC James Hogg Research Centre, Institute for Heart + Lung Health, St. Paul's Hospital, University of British Columbia, Vancouver, British Columbia, Canada; National Heart and Lung institute, United Kingdom

## Abstract

**Objective:**

No optimal housekeeping genes (HKGs) have been identified for CD4^+^ T cells from non-depressive asthmatic and depressive asthmatic adults for normalizing quantitative real-time PCR (qPCR) assays. The aim of present study was to select appropriate HKGs for gene expression analysis in purified CD4^+^ T cells from these asthmatics.

**Methods:**

Three groups of subjects (Non-depressive asthmatic, NDA, n = 10, Depressive asthmatic, DA, n = 11, and Healthy control, HC, n = 10 respectively) were studied. qPCR for 9 potential HKGs, namely RNA, 28S ribosomal 1 (*RN28S1*), ribosomal protein, large, P0 (*RPLP0*), actin, beta (*ACTB*), cyclophilin A (*PPIA*), glyceraldehyde-3-phosphate dehydrogenase (*GAPDH*), phosphoglycerate kinase 1 (*PGK1*), beta-2-microglobulin (*B2M*), glucuronidase, beta (*GUSB*) and ribosomal protein L13a (*RPL13A*), was performed. Then the data were analyzed with three different applications namely BestKeeper, geNorm, and NormFinder.

**Results:**

The analysis of gene expression data identified *B2M* and *RPLP0* as the most stable reference genes and showed that the level of *PPIA* was significantly different among subjects of three groups when the two best HKGs identified were applied. Post-hoc analysis by Student-Newman-Keuls correction shows that depressive asthmatics and non-depressive asthmatics exhibited lower expression level of *PPIA* than healthy controls (*p*<0.05).

**Conclusions:**

*B2M* and *RPLP0* were identified as the most optimal HKGs in gene expression studies involving human blood CD4^+^ T cells derived from normal, depressive asthmatics and non-depressive asthmatics. The suitability of using the *PPIA* gene as the HKG for such studies was questioned due to its low expression in asthmatics.

## Introduction

It has been proposed that a spectrum of psychological conditions such as depressive disorders occurs at high frequencies in asthmatics [1], and are associated with poor control and worse asthma-related quality of life [2], but the underlying pathophysiological mechanisms that account for this relationship have yet to be elucidated [3]. Since the initial studies of the roles of T cells in the pathogenesis of asthma [4], [5], our understanding of the CD4^+^ T lymphocyte in the immunopathology of this disease has greatly advanced over the past decades, involving not only the classic Th1 and Th2 cells, but also new proinflammatory and suppressive T-cell subsets [6]. Meanwhile, accumulating evidence suggests that CD4^+^ T cells may influence susceptibility to depression as well as its treatment outcomes [7]. Thus, the CD4^+^ T lymphocyte is emerging as a potentially attractive cell in which to seek novel insights into the pathogenesis of asthma with or without depression and to identify new therapeutic targets.

The comparison of gene expression profiling of CD4^+^ T cells in asthmatic subjects with and without depressive disorders can lead to the identification of genes implicated in such diseases and provide added insight into the underlying pathophysiological mechanisms. Real-time quantitative PCR (qPCR) is a useful technique for acquiring the gene expression pattern of a number of selected genes due to its high sensitivity, specificity and broad quantification range [8]. To obtain accurate and reliable gene expression quantification, normalization of gene expression data against housekeeping genes (HKGs) is particularly important. For this purpose, an ideal HKG should be either stably expressed across experimental conditions or similarly expressed among samples affected by different disease processes. However, commonly used HKGs vary considerably in different disease processes or different tissue and cell types. Thus, it is important to perform rigorous validation of the most stable HKGs in different tissues or cells and/or disease status before commencement of any qPCR study. Before comparison of gene expression profiling of CD4^+^ T cells in pure asthmatics or depressive asthmatics, this requirement must be met. However, there have been no studies that have systematically compared the stability of common HKGs in such conditions.

In the present study, we carried out a careful evaluation of 9 HKGs in uncultured human CD4^+^ T cells derived from healthy individuals, non-depressive asthmatics and depressive asthmatics. After analysis and comparison using three different statistical methods, *B2M* and *RPLP0* were identified as the most suitable HKGs for gene expression studies in uncultured CD4^+^ T cells of asthmatics with or without depression.

## Materials and Methods

### Patients

Three groups of subjects were studied: asthmatics with depression (Depressive asthmatics, DA), asthmatics without depression, (Non-depressive asthmatics, NDA) and Healthy controls (HC). Patients of DA group (n = 11) and NDA group (n = 10) were enrolled from the outpatient clinic of the West China Hospital of Sichuan University from September 2011 to January 2012 as a cross-sectional study. All patients had symptoms consistent with diagnosis of asthma and demonstrated evidence of bronchodilator reversibility of >12% and 200 mL in forced expiratory volume in 1 s (FEV_1_) following 400 µg of inhaled salbutamol or provocative dose of methacholine causing a 20% drop in FEV_1_ (PD_20_FEV_1_) <2.5 mg. A bronchial challenge test was performed for all of the patients, except one from the NDA group and four from the DA group who had an FEV_1_ predicted less than 70%. Atopic status was determined by a positive skin prick test result using 14 common aeroallergens, including *Dermatophagoides*, animal hair, cockroach, pollens, *Platane*, *Saccharomyces, Penicillium*, cigarette, cotton fibre and feather. All patients answered a detailed respiratory health and general history questionnaire. The depression status was evaluated by the same psychiatrist using the Hamilton Rating Scale for Depression (HRSD) [9], which is observer-rated and a score ≥8 was used as a cut-off point for comorbid depressive symptoms. The clinical control of asthma was assessed with Asthma Control Test (ACT) [10] and health-related quality of life was appraised with the Standardized Version of the Asthma Quality of Life Questionnaire (AQLQs) [11]. HC subjects (n = 10) with no significant medical conditions were recruited by advertisement.

Subjects were excluded if they had been taking oral glucocorticosteroids within 4 weeks of the blood draw. Other exclusion criteria included uncontrolled asthma or change in maintenance therapy, acute respiratory tract infection within 4 weeks, and current smoking. The present study received approval from the West China Hospital Institutional Review Board and all participants gave written informed consent.

### Purification of CD4^+^ T lymphocytes from Adult Blood

**Figure 1:**
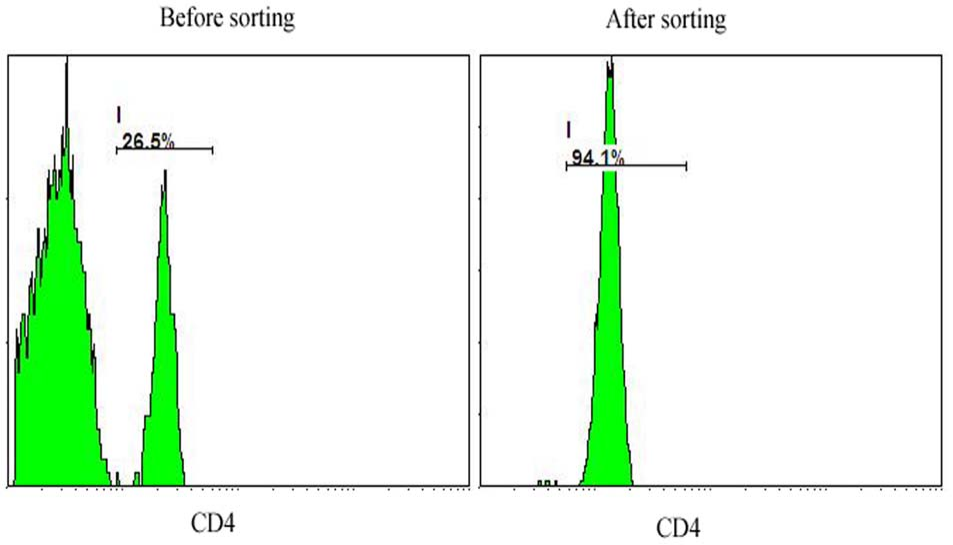
Flow cytometry analysis on CD4^+^ T cells in lymphocyte suspension and in purified CD4^+^ cells by immunomagnetic depletion with the human CD4^+^ T Cell Isolation Kit II. Cells were directly stained with conjugated fluorescently labeled antibodies for CD4 (BD Biosciences) in lymphocyte suspension and in purified CD4^+^ samples.

We obtained up to 10 mL whole blood from each subject and specimens were shipped from the clinic to a processing laboratory within 1 hour of collection and handled in exactly the same manner by the same technician. Lymphocyte suspensions were separated by Lymphoprep (Axis-Shield PoC AS, Oslo, Norway) from a distinct band at the sample interface. CD4^+^ T Lymphocytes were purified by immunomagnetic depletion with the human CD4^+^ T Cell Isolation Kit II (Miltenyi Biotec, Rostock, Germany). The mean ± SD number of total lymphocytes was 22.87±5.68 million. After separation, each sample yielded ∼2.4 million CD4^+^ T cells and 1 million cells were used for RNA extraction from each sample. Furthermore, our pilot studies have confirmed that the CD4^+^ T cell population isolated by this method has a purity of over 94%, which was shown by ﬂow cytometry (Figure 1).

### Selection of Reference Gene Candidates

Eleven HKGs from the endogenous control panel genes recommended by Applied Biosystems (http://www.appliedbiosystems.com/) were initially selected. 18 S ribosomal RNA (*RNR1*) was replaced by RNA, 28 S ribosomal 1 (*RN28S1*) due to their stable expression ratio in integrated RNA samples and the availability of *RN28S1* assay in our laboratory. Ribosomal protein L13a (*RPL13A*) was added because it was a stable expression gene in CD4^+^ cells from a previous study [12]. Among of the 12 genes selected, hypoxanthine ribosyltransferase (*HPRT1*), TATA-binding protein (*TBP*) and transferrin receptor (*TFRC*) have low expression levels in the CD4^+^ cells and whole blood therefore they were omitted from the final list (http://www.genecards.org/).

Nine housekeeping genes were examined, including *RN28S1*, ribosomal protein, large, P0 (*RPLP0*), actin, beta (*ACTB*), cyclophilin A (*PPIA*), glyceraldehyde-3-phosphate dehydrogenase (*GAPDH*), phosphoglycerate kinase 1 (*PGK1*), beta-2-microglobulin (*B2M*), glucuronidase, beta (*GUSB*), and *RPL13A*. The full name, function and accession number of the candidate HKGs evaluated in the present study are listed in Table 3. Special attention was paid to selecting candidate genes that show a diversity of function, which significantly reduces the chance that genes might be co-regulated.

### RNA Extraction and Complementary DNA Preparation

Total RNA was isolated using Trizol (Invitrogen, Carlsbad, California, USA) following the manufacturer’s protocol. RNA integrity was assessed on the basis of demonstration of distinct 28 s and 18 s ribosomal RNA bands following 1% agarose electrophoresis and the 28 S RNA was approximately twice as intense as the 18 S rRNA. Complementary DNA (cDNA) synthesis was carried out using the RevertAid™ first strand cDNA synthesis kit (Fermentas Inc, Burlington, Canada). Template RNA and 1 µL of random hexamer primers (10 µM) in a total volume of 12 µL were incubated for 5 min at 65°C and chilled on ice. After adding 4 µL of 5×reaction buffer, 1 µL of RiboLock™ RNase Inhibitor (20 U/µL), 2 µL of dNTP Mix (10 mM), 1 µL of RevertAid™ M-MuLV Reverse Transcriptase (200 U/µL), the incubation step for 5 min at 25°C, followed by reverse transcriptase incubation for 60 min at 42°C, termination of the reaction by heating at 70°C for 5 min, finally cooling to 4°C before storage at −20°C. The cDNA for assays of *ACTB*, *GAPDH*, *B2M*, *PPIA* and *RPLP0* was diluted 1∶25 because these genes were highly expressed in pilot studies; while assays of *RN28S1*, *GUSB*, *RPL13A* and *PGK1* were performed using cDNA diluted 1∶15 because they had relatively low expression levels.

### Real-time Quantitative PCR

The expression analysis for all 9 genes was performed using an FTC 2000 qPCR system (Funglyn Biotech Inc, Scarborough, Canada), PCR primers and TaqMan probes were obtained from Shanghai biological engineering corporation, China (see Table 4 for primer sequences). The reactions were performed according to the manufacturer’s instructions with minor modifications. Briefly, 2 µL template cDNA was used in a final PCR reaction volume of 30 µL, containing 0.3 µL of 5 U/µL Taq DNA polymerase, 3 µL of 25 mM MgCl_2_, 0.36 µL of 25 mM dNTP, (TAKARA Bio Group, Dalian, China), 1 µL of 10 µM of each forward and reverse primer and probe. The conditions for the PCR included for 2 min at 94°C followed by 45 cycles of real-time PCR with 3-segment amplification, including 20 s at 94°C for denaturation, 20 s at 52°C (*RN28S1*, *PPIA*, *GAPDH* and *RPL13A*), 54°C (*RPLP0*, *ACTB*, *GUSB*) or 56°C (*PGK1* and *B2M*) for annealing, and 30 s at 60°C for polymerase elongation. All reactions were performed in triplicate, with non-template controls and standard curves which were generated using four serial dilution points (in steps of 10-fold) of stock cDNAs for each gene. The threshold cycle (Ct) was manually determined from amplification plots. The ΔCt value for each sample was obtained by subtracting the Ct values of the highest relative quantities for each gene, and was converted into relative gene expression by the amplification efficiency (2 = 100%) to the −ΔCt power.

### Statistical Analysis

In order to identify the optimal reference genes among the candidates, three different tools called BestKeeper, geNorm, and NormFinder based on specific algorithms were used. The BestKeeper [13] and geNorm [14] determines the optimal HKGs by performing similar pair-wise correlation approach. The NormFinder produces a comparison of the rankings by a model-based approach and focuses on estimating both the overall variation of the reference genes and the variation between subgroups [15].

Clinical data are reported as mean ± SD for normally distributed data and median (range) for nonparametric data. Descriptive statistics of the 8 HKGs were computed by BestKeeper.

The comparisons of gene expression levels and demographic characteristics of the participants between subgroups were performed by using the one-way ANOVA (two tailed) for parametric data, Kruskal-Wallis *H* test for nonparametric data and Student-Newman-Keuls test for multiple comparisons. All analyses were conducted with SPSS software, version 18.0 (IBM Corp, New York, USA). *P*<0.05 was considered significant.

## Results

### Subjects

Characteristics of the three groups of participants are summarized in Table 1. By design, all 3 groups (NDA, n = 1, DA, n = 11, and HC, n = 10) were similar in sex and age distribution. All subjects were non-smokers or former smokers and there were only two former smokers, one in NDA group and the other in NC group. Both of them have quitted at least 10 years and had smoked cigarette 4.5 and 0.5 pack-years, respectively. All medications were discontinued for a minimum of 2 weeks before recruitment. In detail, one patient from the NDA group and two from DA group inhaled inhaled corticosteroid (ICS) + Long-acting β_2_-agonists (LABA). However, all of them used ICS + LABA for a maximum of 1 month and discontinued at least 4 weeks before blood was drawn. Four patients from the NDA group and three from the DA group took theophylline, and one patient from each group took antileukotrienes orally. However, the medications were discontinued at least 2 weeks before the experiment.

**Table 1:** Demographic characteristics of the participants.

Clinical characteristic	Non-depressive asthmatics	Depressive asthmatics	Healthy controls
**N**	10	11	10
**Sex, male:female**	3∶7	2∶9	2∶8
**Age (y)** a	33.70±10.58	34.18±9.41	30.6±7.41
**BMI, kg/m^2a^**	23.10±2.50	22.26±2.28	22.13±3.25
**Ex-smoker, n (%)**	1 (10)	0 (0)	1 (10)
**Non-smoker, n (%)**	9 (90)	11 (100)	9 (90)
**Atopy, n (%)**	7 (70)	4 (36.4)	4 (40)
**FEV_1_% predicted** a,c	91.12±15.69	82.28±17.07	116.86±15.21
**FVC% predicted** a,d	97.31±11.20	95.55±11.36	107.64±14.28
**FEV1/FVC%** a,c	79.29±8.90	74.23±11.10	88.38±5.90
**PD_20_FEV_1_ Methacholine (mg)** b	0.50 (0.08–2.47)	0.94 (0.31–2.30)	–
**ACT score** a	16.10±4.84	14.91±2.70	–
**Anaphylactic history, n (%)** c	8 (80)	6 (54.5)	1 (10)
**GINA severity**			
Mild, n (%)	1 (10)	0 (0)	–
Moderate, n (%)	5 (50)	4 (36.4)	–
Severe, n (%)	4 (40)	7 (63.6)	–
**AQLQ** a	4.45±0.87	3.96±0.68	–
**Eosinophils (10^9^/L)** b	0.24 (0.06–0.57)	0.20 (0.06–1.22)	0.15 (0.02–0.49)
**Eosinophils (%)** b	4.35 (1.10–10.5)	5.10 (1.6–14.1)	2.45 (0.3–11.6)
**Total IgE (IU/ml)** b,c	190.88 (34.17–1002.29)	63.82 (0.05–547.81)	44.41 (8.11–189.05)
**Asthma medication used in past year (discontinued at least 2 weeks before recruitment)**			
ICS + LABA, n (%)	1 (10)	2 (18.18)	–
Theophylline, n (%)	4 (40)	3 (27.27)	–
Antileukotrienes, n (%)	1 (10)	1 (9.09)	–

a mean ± SD;

b median (range);

c *P*<0.05;

d *P* = 0.073; BMI, body mass index; FEV_1_, forced expiratory volume in 1 s; FVC, forced vital capacity; ACT, Asthma control test; GINA, Global initiative for asthma; AQLQ, Asthma quality of life questionnaire; Ig, immunoglobulin; PD_20_FEV_1_, provocative dose of methacholine causing a 20% drop in FEV_1_; ICS, inhaled corticosteroid; LABA, Long-acting β_2_-agonists.

There were significant differences between subgroups in FEV_1_% predicted, FEV_1_/forced vital capacity (FVC) %, the proportion having anaphylactic history and total immunoglobulin E (IgE) present in each sample. Age, sex, body mass index (BMI), the proportion of participants who were atopic, number of eosinophils and the proportion of eosinophils did not differ among the three groups. There were no significant differences in demographic characteristics such as PD_20_FEV_1_ and asthma severity, etc. between NDA and DA groups (see Table 1 for detail).

### Expression Levels of Candidate HKGs

Nine HKGs (n = 31) were investigated and triplicate assays were performed for each of the 31 subjects. The Ct values were over 45 for 5 samples for *GAPDH* and therefore this gene was excluded from further analysis. For the remaining 8 HKGs, two samples had three unidentified Ct values in assays for two genes: one sample from the HC group and the other from the DA group. Thus both of these samples were excluded from further analysis. One of the specimens investigated exhibited 3-fold intrinsic variance (InVar) compared with the mean InVar of all HKGs, therefore, this subject was excluded. The final analysis contained 28 samples for 8 HKGs (Table 2).

**Table 2:** Data of candidate housekeeping genes (n = 28).

	*RN28S1*	*RPLP0*	*ACTB*	*PPIA*	*PGK1*	*B2M*	*GUSB*	*RPL13A*
**n**	28	28	28	28	28	28	28	28
**GM**	14.90	26.69	26.37	27.54	28.63	24.36	32.54	25.99
**AM**	14.94	26.72	26.39	27.57	28.67	24.38	32.56	26.02
**min**	12.83	23.77	23.83	23.93	24.33	22.33	30.07	24.00
**max**	16.46	28.23	28.65	29.82	31.10	26.49	35.22	28.43
**SD**	0.92	0.97	1.02	0.89	1.20	0.88	0.83	0.95
**CV (%)**	6.19	3.65	3.85	3.22	4.19	3.63	2.56	3.66

GM, geometric mean; AM, arithmetic mean; Min, minimal value; Max, maximal value; CV, coefficient of variance.

**Table 3:** Housekeeping genes evaluated in the present study.

Full name	Symbol	Gene function	Accession no.
RNA, 28S ribosomal 1	*RN28S1*	Riboxomal units	ENST00000419932
Ribosomal protein, large, P0	*RPLP0*	Structural component of the 60S subunit of ribosomes	NM_001002.3
Actin,beta	*ACTB*	Cytoskeletal structural actin	NM_001101
Cyclophilin A	*PPIA*	Accelerate the folding of proteins	NM_021130.3
Glyceraldehyde-3-phosphate dehydrogenase	*GAPDH*	Enzyme in glycolysis and nuclear functions	NM_002046
Phosphoglycerate kinase 1	*PGK1*	Glycolytic enzyme	NM_000291.3
Beta-2-microglobulin	*B2M*	Component of the major histocompatibility complexclass I molecules	NM_004048.2
Glucuronidase, beta	*GUSB*	Hydrolase that degrades glycosaminoglycans	NM_000181.3
Ribosomal protein L13a	*RPL13A*	Structural component of the 60S ribosomal subunit	NM_012423.2

### Expression Stability within HKGs

**Figure 2:**
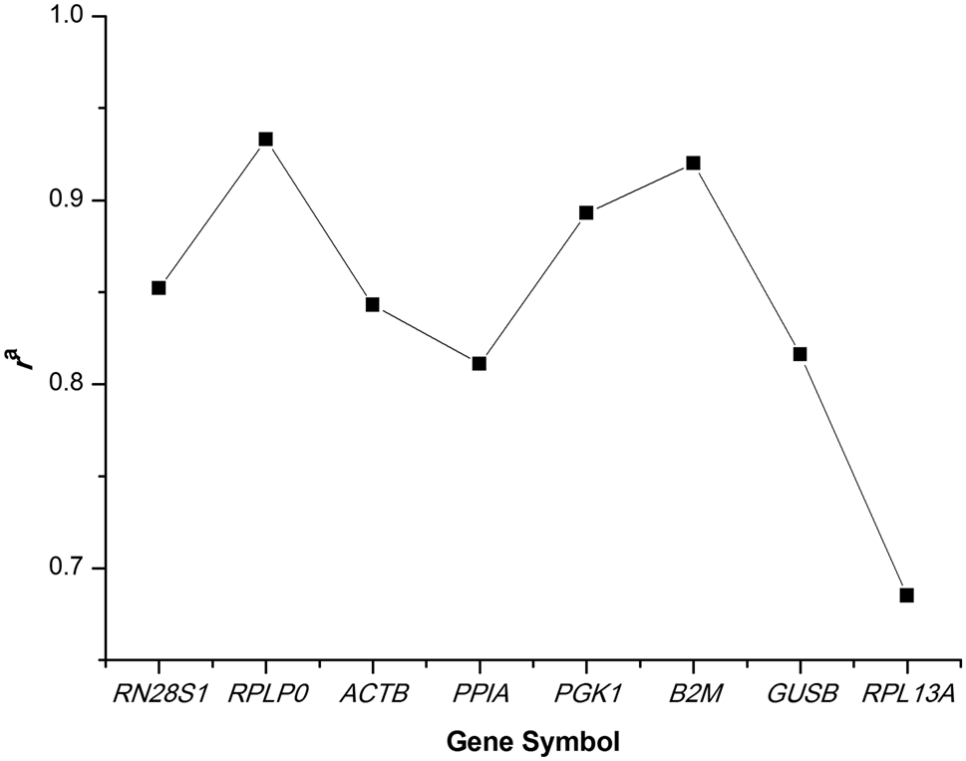
Correlation analysis of candidate housekeeping genes (HKGs) versus BestKeeper index. ^a^All 8 candidate HKGs showed strong correlation (correlation coefficient *r* value from 0.69 to 0.93) and were combined into an index, which was then used to compute the correlation between each HKG and the index.

HKG stability was evaluated using three different Excel-based tools, BestKeeper, geNorm and NormFinder. The BestKeeper was used to rank the candidates’ stability by performing a pair-wise comparative analysis across HKGs. All 8 candidate HKGs showed strong correlation (0.69<*r*<0.93) and were combined into an index. Subsequently, the correlations between each HKG and the index were computed. The highest Pearson correlation coefficient (*r*) value for the relationship between the index and the contributing HKGs was obtained for *RPLP0* (*r = *0.93, *P = *0.001) (Figure 2).

**Table 4:** Primer sequences for housekeeping genes.

Symbol	Forward primer	Reverse primer
*RN28S1*	CTCCCACTTATTCTACACCT	CCACTGTCCCTACCTACTAT
*RPLP0*	CTGGAAGTCCAACTACTTCCT	CATCATGGTGTTCTTGCCCAT
*ACTB*	GAAGATCAAGATCATTGCTCCT	TACTCCTGCTTGCTGATCCA
*PPIA*	TCCTGGCATCTTGTCCAT	TGCTGGTCTTGCCATTCCT
*GAPDH*	AAGCTCATTTCCTGGTATGACA	TCTTACTCCTTGGAGGCCATGT
*PGK1*	GCCACTTGCTGTGCCAAATG	CCCAGGAAGGACTTTACCTT
*B2M*	CTATCCAGCGTACTCCAAAG	GAAAGACCAGTCCTTGCTGA
*GUSB*	CCAGTTTGAGAACTGGTATAAG	CTGGTACTCTTCAGTGAACAT
*RPL13A*	CTTTCCTCCGCAAGCGGAT	CCACCATCCGCTTTTTCTT

**Figure 3:**
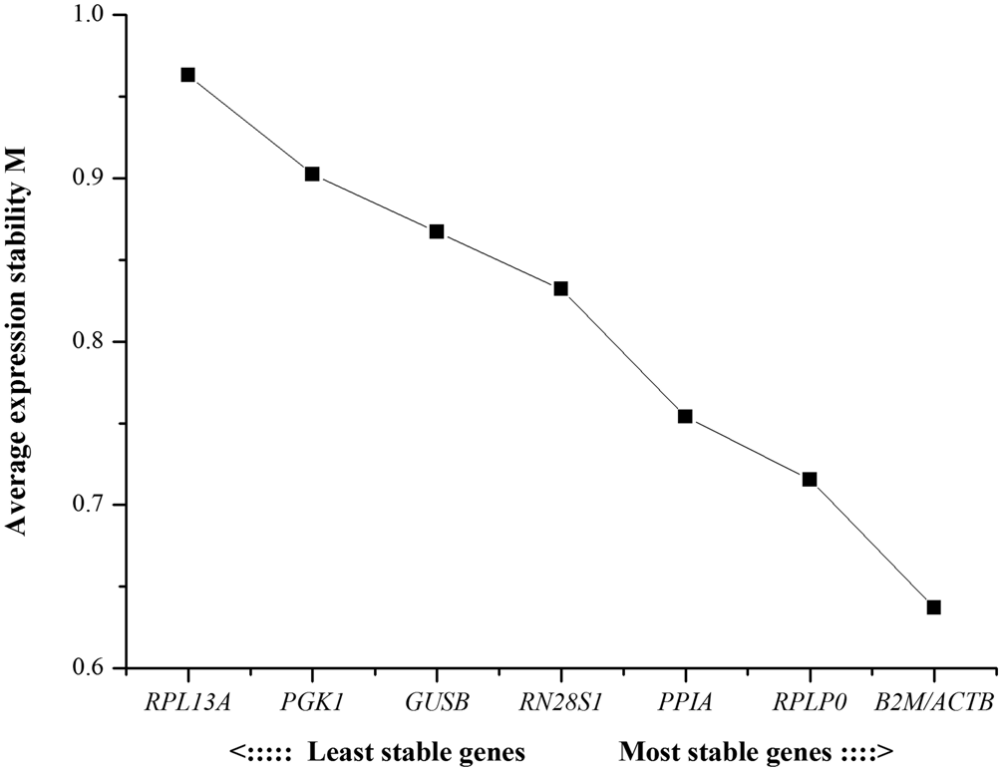
Ranking the housekeeping genes (HKGs) according to their expression stability M determined using geNorm. A stepwise exclusion of the least stable HKG was conducted to obtain the mean expression stability value M of remaining HKGs until the two most stable HKGs were identified. The genes are ranked according to M values.

The geNorm applet calculates a gene expression stability M based on the geometric average V between all tested genes. All 8 studied genes had M values below the default limit of 1.5, which demonstrated that all genes tested had high expression stability. After stepwise exclusion of the worst-scoring HKGs, *B2M* and *ACTB* were identified as the two most stably expressed genes in the studied samples (Figure 3).

**Figure 4:**
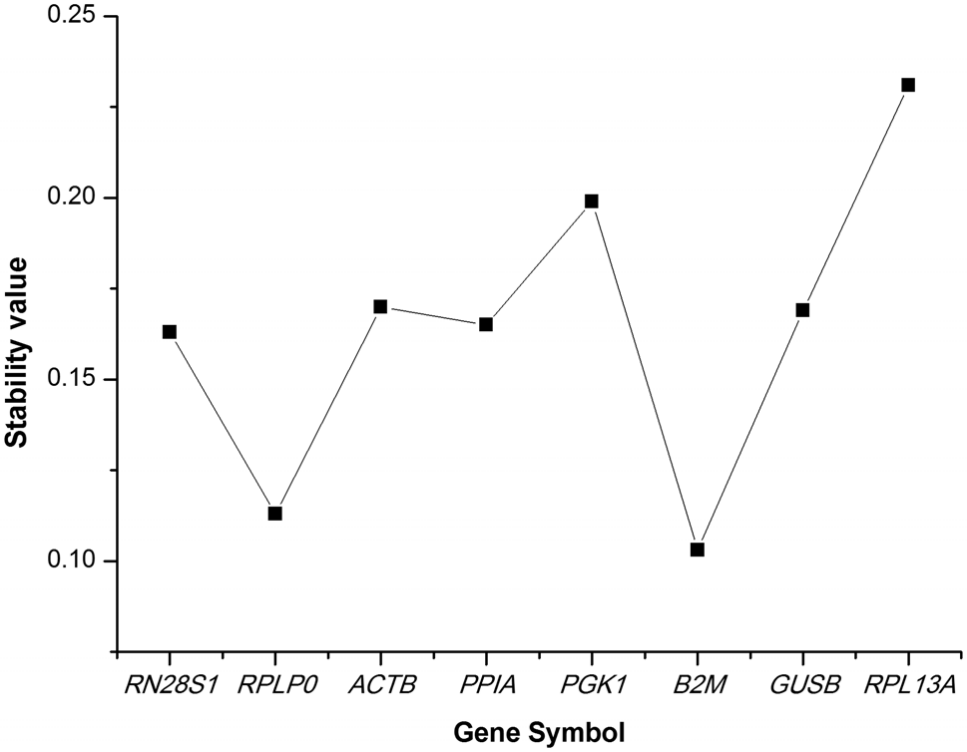
Determination of the housekeeping gene expression stability by NormFinder. The stability value is estimated using the model-based approach. Having considered both the intra- and inter-group variation, a lower stability value represents a smaller systematic error that would be introduced when using the studied gene.

Finally, the NormFinder program was used to rank candidate HKG stability. This applet uses a model-based approach to estimate the intragroup and intergroup expression variation, and then combines them into a stability value that makes ranking the candidate genes across different disease status possible. The best gene identified by this program was *B2M* (Figure 4), and the best combination of two genes was *B2M* and *RPLP0*. Although the order of stability of the studied genes differed slightly among the 3 applets, the top 2 ranked genes exhibited similarly.

### Expression Levels of Candidate HKGs in Three Groups of Subjects

**Figure 5:**
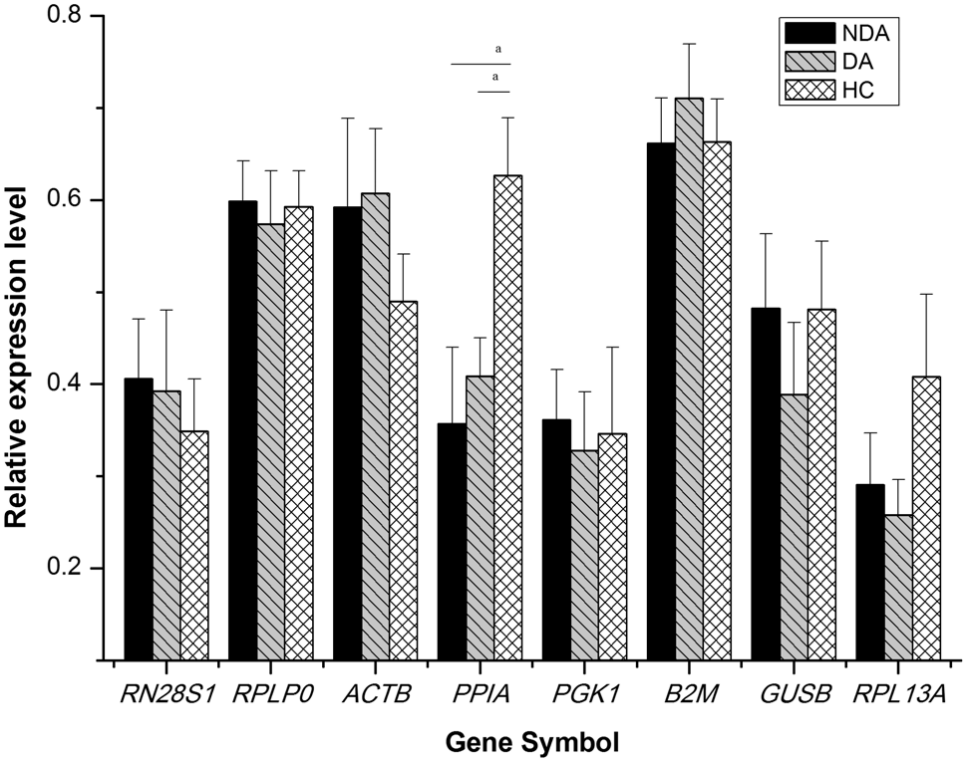
Comparison of the normalized relative expression levels of housekeeping genes (HKGs) between the three subgroups. The relative expression levels of remaining seven genes were normalized against the Normalization Factor based on the geometric mean of the expression level of the best-performing HKGs (*B2M* and *RPLP0*). Data are presented as mean ± SE. ^a^ *P*<0.05.

Both *B2M* and *RPLP0* were the top two most stable HKGs as generated by the three different analytical methods. Thus, a normalization factor (NF) based on the geometric mean of the expression level of the best-performing HKGs was calculated by geNorm to compare the expression levels of the other candidate HKGs in the three subgroups. The results show that, before normalization, the expression levels were not significantly different among the three groups for all candidate genes. After normalization, the expression levels of *PPIA* were significantly different between the three groups; Post hoc analysis by Student-Newman-Keuls test shows that depressive asthmatics and non-depressive asthmatics exhibited lower expression levels of *PPIA* than healthy controls (*p*<0.05) (Figure 5).

## Discussion

The literature [6] overwhelmingly confirms that CD4^+^ T cells play an important role in the occurrence and development of asthma, and an increasing amount of evidence [7], [16] supports the concept that these cells also influence susceptibility to depression. The results of the present study will enable meaningful interpretation of data from expression studies that use purified CD4^+^ T cells, and therefore can help understanding of underlying mechanisms.

Real-time quantitative PCR is a routinely used technique to measure transcript abundance with great sensitivity, specificity and reproducibility. Nevertheless, exact normalization of gene expression levels is an absolute prerequisite for reliable results of qPCR quantification methods.

This study demonstrates the use of three different Excel-based applets to identify the most stable HKGs in the studied population. Expression stability for a single sample or each HKG was investigated using BestKeeper first. All of the studied 28 samples had low InVar fold level. An InVar value of more than 3-fold indicates low consistency and reliability. The geNorm applet uses a pairwise comparison approach similar to BestKeeper to identify the best combination of two genes based on the geometric mean expression levels [15]. However, it uses the transformed expression levels instead of raw Ct data used in BestKeeper to control the profound influence made by any outliers.

The NormFinder uses a model-based approach to provide a more precise measure of gene expression stability due to its direct estimation of expression variation and consideration of systematic differences between subgroups, rather than pairwise comparison approach [14]. In addition, the pairwise comparison approach is probably influenced by HKG co-regulation, and therefore the final ranks may not be optimal.

*PPIA* encodes a member of the peptidyl-prolyl cis-trans isomerase (PPIase) family, which are ubiquitous intracellular proteins that play a role in cyclosporine A-mediated immunosuppression [17]. The role of PPIA in allergic asthma is inconsistent in the literature. On one hand, PPIA^−/−^ lockout mice developed allergic disease accompanied by elevated IgE and an increased number of mast cells and eosinophils in multiple tissues, which was caused by type 2 cytokines released from CD4^+^ T cells [18]. While on the other hand, increasing evidence has suggested that cyclophilins are potent chemoattractants for a variety of human and mouse leukocyte subsets [19], [20]. Indeed, elevated protein levels of cyclophilin have been observed both in acute allergic asthma [21] and chronic periods of the disease. Blocking the function of PPIA reduced the recruitment of leukocytes and acute episodes of the disease following allergen challenge [22]. In the present study, PPIA mRNA level was lower in asthmatics than in healthy controls. One explanation is that in the present study, unstimulated CD4^+^ T cells were studied. It is possible that *PPIA* level is low in resting CD4^+^ T cells. Upon allergen stimulation, such as in acute asthmatics or chronic asthmatics with continuous allergen exposure, *PPIA* expression would be higher than normal. This phenomenon was seen previously with other chemoattractants such as eotaxin, RANTES, MIP-1α, and MCP-1 [23], [24], [25]. Our previous study identified *PPIA* as a stable expressed HKG in airway epithelial cells [26], this paper has provided helpful information to a dozen of studies since its publication (citations from Google Scholar). Several publications used *PPIA* as a HKG to normalize the expression levels of target genes and found meaningful differential expressions of target genes [27], [28], Current study identified *B2M* and *RPLP0* as the most optimal HKGs in gene expression studies involving human blood CD4^+^ T cells derived from normal subjects and asthmatics with and without depression. The different results from the two studies may be explained by the fact that the cell types in the two studies were different and our results have also strengthened the importance of optimal HKGs selection before performing any qRT-PCR in different disease conditions. Since asthma with depression have been considered to influence the disease process of asthma certainly, exploring the underlying pathophysiological mechanisms is necessary. However, before we determine the molecular basis, selecting optimal HKGs is the first and crucial step.

### Conclusions

To our knowledge, this is the first study to identify the most stable HKGs in CD4^+^ T cells and depressive/non-depressive asthmatic disease status. *B2M* and *RPLP0* were identified as the most optimal combination of HKGs in gene expression studies involving human blood CD4^+^ T cells derived from normal, depressive asthmatics and non-depressive asthmatics. Moreover, the present findings question the suitability of the *PPIA* gene as the HKG for such studies due to its significantly lower expression levels in asthmatic CD4^+^ T cells. Furthermore, careful comparison of the gene expression profiles of purified CD4^+^ T cells based on information from this study will further elucidate the molecular basis of the incidence and development of asthma with or without depression.
